# 

**Published:** 1914-03-28

**Authors:** 


### HOSPITAL CHAIRMAN'S WILL.

Mr. Ernest Arthur Lazarus-Barlow, London manager and a director of the Comptoir National d'Escompte de Paris, a director of the Anglo-Argentine Tramways Company (Ltd.), a former chairman of the British Thomson-Houston Company (Ltd.), and chairman of the French Hospital in London, has left estate valued at £226,332 gross, with net personalty £221,315.

THE
### Doctors' Wives Association.
(*See page* 695.)

**COUPON.**

I hereby certify that I am a doctor's wife, and I beg to send you my name and address as one who wishes to become a member of the proposed Doctors' Wives Association. I hereby agree to qualify as a member by paying the subscription of 7s. 6d. per annum on or before May 1, 1914, such subscription entitling me each year to a free copy of The Hospital newspaper delivered weekly, with the Doctors' Wives section, and to include my annual subscription to the Association.

This agreement undertaking to subscribe on my part is subject to at least 1,000 doctors' wives conditionally joining the proposed Association by signing and returning to "Co-operation" as below by April 18 next a similar coupon to that to which I hereby attach my signature.

(Signed) ..........................................

(Wife of) * ..........................................

(Address) ..........................................

..........................................

(Date) ..........................................

*Note.*—When filled in and signed cut out this coupon, place it in an envelope and address and post it to :

Co-Operation,
c/o The Scientific Press,
28 & 29 Southampton Street, Strand,
London, W.C.

* Here insert full name and qualifications.

### Appointments.

The following appointments have been made at the Manchester Royal Infirmary :—Honorary assistant physicians : F. E. Tylecote, M.D., Ch.B., D.P.H.(Vict.), M.R.C.P.(Lond.), and C. H. Melland, M.D.(Lond.), B.Sc.(Vict.), M.R.C.P.(Lond.). Honorary assistant surgeons : W. H. Hey, M.B., Ch.B.(Vict.), F.R.C.S.(Eng.), L.R.C.P.(Lond.), and J. P. Buckley, M.A., M.D., B.C.(Cantab.), M.B., M.S.(Lond.), F.R.C.S.(Eng.), L.R.C.P.(Lond.). Honorary dental surgeon : E. G. Long, L.D.S.(Eng.).

Dr. John A. Edmond, M.B., B.S., has been appointed by the Lambeth Board of Guardians third assistant medical officer at the infirmary in the place of Dr. Rees Phillips, who has resigned. Dr. P. L. Watkin, M.R.C.S., L.R.C.P., who has acted as *locum tenens*, has been appointed fourth medical officer at the infirmary.

Mr. E. Treacher Collins, F.R.C.S., has been re-appointed by the Metropolitan Asylums Board as their ophthalmic surgeon. He has held this post since November 1901, and receives an annual remuneration of £360 for the two schools. At present he inspects the children in London before they are sent to the schools, visits each school twice in three weeks, and certifies every child before discharge. The Board has decided that Mr. Collins shall in future visit each school once a fortnight instead of once every ten days, and, as a set-off against the reduction in the number of visits, he will see any eye cases presented to him at his examinations in London from any other institutions of the Board which the children's committee report to be very desirable, but which has not been included in the scope of his duties at present.

Mr. P. O'C. Finigan, L.D.S. R.C.S. (England), D.D.S. (University of Michigan), has been appointed by the Metropolitan Asylums Board dentist for the institutions under the control of the children's, asylums, and *Exmouth* committees. It is a whole-time appointment. Mr. Finigan is at present in private practice, and for the past five years has held the appointment of dental surgeon to the staff of the Civil Service Co-operative Society. Previously he was dental house surgeon at the Dental Hospital.

Dr. H. R. Wilson, M.D., M.B., B.Sc., M.R.C.S., L.R.C.P., of Merthyr Tydfil, tuberculosis physician to the Welsh National Memorial to King Edward VII., has been appointed by the Southwark Borough Council tuberculosis officer for the borough. Dr. Wilson was also consulting physician to the tuberculosis pavilions of Merthyr Tydfil and Aberdare Valleys Branch of the Welsh National Memorial and physician to the Merthyr Borough Insurance Committee and Glamorgan County Insurance Committee. His previous appointments include those of resident medical officer at the Royal Infirmary, Liverpool; consulting physician and organiser of the tuberculosis pavilions at the National Memorial, Merthyr Isolation Hospital; resident physician at Crookesbury Sanatorium; clinical assistant and deputy out-patient physician at Mount Vernon Hospital; and out-patient physician at City Road Chest Hospital. He is a member of the National Association for Prevention of Consumption, and has written articles on tuberculosis. Dr. Wall, of the Brompton Hospital, has been thanked by the Council for the services he had rendered in the selection of candidates.

---

The Mental Hospitals Committee of the London County Council have recently granted permission to Dr. Bonnichsen, a doctor of medicine of Copenhagen, to work as a resident clinical assistant at Hanwell Mental Hospital for a period of two months without salary.

### Gifts and Bequests.

The visit of the King and Queen to the Palladium Theatre on Tuesday last week has added £2,000 to the fund for rebuilding the Chelsea Hospital for Women.

Mr. Arthur William Walker, of Leominster, Hereford, has bequeathed £1,000 to the Hereford General Hospital and £500 to the Leominster Cottage Hospital.

Miss Jane Barbara Thomas, late of Bath and of Dublin, has left to Dr. Barnardo's Homes a gold ring with an elephant's hair, which was sent by her brother from Ceylon.

Mr. Benjamin Lewtas, of Hambleton, Lancs, yeoman, has left £250 each to the Preston and County of Lancashire Royal Infirmary, the Royal Albert Asylum, Lancaster, Victoria Hospital, Blackpool, Fleetwood Cottage Hospital, and Dr. Barnardo's Homes.

Mr. Carew Davies Gilbert, of Truro and Eastbourne, has bequeathed £500 each to the Royal Cornwall Infirmary, Princess Alice Hospital, Eastbourne, and King Edward's Hospital Fund; and he empowered his trustees in their discretion to continue for one year his subcriptions to charitable institutions.

Mr. Alexander Allan, of Aberdeen, has left £10,000 to the Aberdeen Royal Infirmary; £5,000 to the Aberdeen Royal Sick Children's Hospital; £4,000 to the Morningfield Hospital, Aberdeen; and £2,000 to the Aberdeen Convalescent Hospital. Subject to several other bequests, the residue of his property to six institutions, including those already mentioned, in the proportion of the legacies named.

Rates of Subscription: Twelve months, 6/6; Six months, 4/-; Three months, 2/-, post free to any address in the United Kingdom. Abroad, Twelve months, 8/8; Six months, 5/-; Three months, 2/6, post free.—Address The Subscription Dept., "The Hospital," The Hospital Building, 28/29 Southampton Street, Strand, London, W.C.

